# Significance of sTREM-1 and sST2 combined diagnosis for sepsis detection and prognosis prediction

**DOI:** 10.1515/biol-2022-0639

**Published:** 2023-08-17

**Authors:** Yongjun Wei, Ping Xiao, Benjuan Wu, Fuxi Chen, Xiaofeng Shi

**Affiliations:** Department of Emergency, Tianjin First Central Hospital, Tianjin, 300192, China; Department of Emergency, Tianjin Beichen Hospital, Tianjin, 300400, China

**Keywords:** diagnosis, sepsis, biomarker, soluble triggering receptor expressed on myeloid cells-1, soluble suppression of tumorigenicity 2

## Abstract

The diagnosis of sepsis still lacks a practical and reliable gold standard. The purpose of this study was to confirm the effect of soluble triggering receptor expressed on myeloid cells-1 (sTREM-1) combined with soluble suppression of tumorigenicity 2 (sST2) in the diagnosis of sepsis through the correlation between sTREM-1, sST2, and sequential organ failure assessment (SOFA) scores. Baseline data of 91 patients with sepsis in the intensive care unit were collected, sTREM-1 and sST2 were detected, and the correlation between markers and SOFA score was analyzed. Besides, the prognostic value of baseline and postadmission indicators for sepsis was analyzed with death as the outcome. The results showed that the expressions of sST2 and sTREM-1 in death group and survival group were higher than those in the survival group (*p* < 0.05). Correlation analysis showed that sST2, sTREM-1, and the joint diagnosis model had a high correlation with SOFA score (*p* < 0.05), but poor correlation with Acute Physiology and Chronic Health Evaluation Ⅱ score (*p* > 0.05). Among them, joint diagnosis model has the highest correlation. Receiver operating characteristic curve analysis showed that combined diagnosis had higher area under curve values. sTREM-1/sST2 can be better used in the diagnosis of sepsis than the single biomarker detection, and the combination of the above two biomarkers has potential application value in the detection and prognosis prediction of sepsis.

## Graphical abstract

**Figure:**
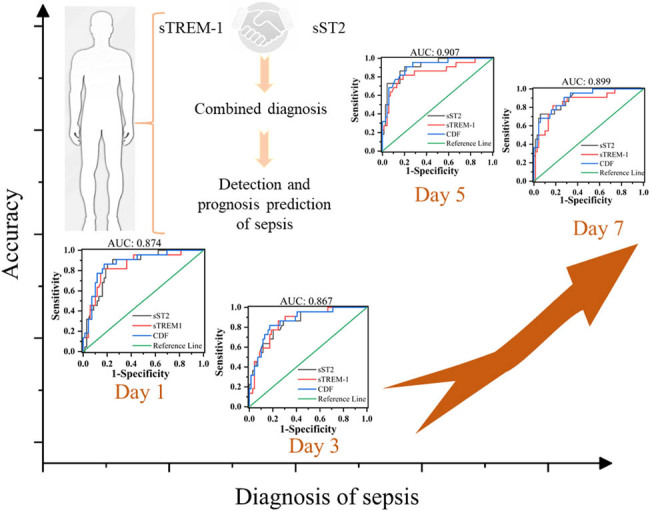

## 1 Introduction

Sepsis is an immune dysfunction of the host caused by a microbial infection that leads to fatal organ and tissue damage [1,2]. When sepsis occurs, the imbalance of systemic inflammatory response leads to vascular endothelial function injury, secondary coagulation dysfunction, and eventually multiple organ dysfunction syndrome [3]. Despite significant advances in the diagnosis and treatment of sepsis, it remains a major cause of life-threatening illness worldwide [4], resulting in 11 million deaths in 2017 alone [5].

In China, the guideline-recommended diagnostic criteria for sepsis are infection or suspected infection with a sequential organ failure assessment (SOFA) score increase of ≥2 points from baseline [6,7]. However, clinical trauma, severe pancreatitis, acute autoimmune diseases, and other non-infectious diseases can also cause the rise of SOFA [8], but the treatment and prognosis of these diseases are completely different. At the same time, diagnosis of sepsis requires baseline and at least one follow-up SOFA evaluation, and waiting for evaluation may result in delayed treatment [9]. In addition, while waiting for the results of microbial culture, doctors often use antibiotics according to experience, increasing unnecessary medical costs and easy to cause bacterial drug resistance, double infection, and other problems. Therefore, timely detection and diagnosis of sepsis is extremely important. However, as nearly half of the infected patients have negative microbial cultures, there is still no practical and reliable gold standard for the diagnosis of sepsis [10]. Therefore, it is important to develop simple and reliable diagnostic methods.

The most commonly used laboratory parameters for sepsis biomarkers include C-reactive protein, procalcitonin (PCT), and lactic acid; cytokines: interleukin (IL)-1β, IL-6, IL-10, and TNF-α, etc. Immune cells and surface receptor markers of innate immune system: soluble leukocyte differentiation antigen 14 subtypes (sCD14-ST), soluble triggering receptor expressed on myeloid cells-1 (sTREM-1), CD64, etc. [11,12]. Although numerous studies have explored biomarkers for the diagnosis of sepsis, it has been found that single cytokines or receptors of immune cells are unlikely to be good biomarkers for sepsis. The sensitivity and specificity of sepsis detection still need to be further improved. Searching for appropriate combinations may provide a better choice for sepsis diagnosis. Besides serving as a diagnostic biomarker, sTREM-1 may also help predict the prognosis of patients with sepsis by measuring its concentration [13,14]. A few studies have proved the feasibility of soluble suppression of tumorigenicity 2 (sST2) to predict the prognosis of sepsis [15,16]. Studies have shown that combined diagnosis has a stronger advantage in the diagnosis of sepsis when the diagnostic accuracy of a single marker is insufficient [17,18,19]. The diagnosis of sepsis by biomarkers sST2 and sTREM-1 is still controversial. We hypothesize that the combined measurement of sST2 and sTREM-1 may contribute to the early diagnosis, severity stratification, and prognosis prediction of patients with suspected sepsis. And, to the best of our knowledge, this is the first time that the two combined measurements have been used in sepsis diagnosis and prognostic prediction.

A series of confirmatory experiments were conducted to verify the effect of sTREM-1 and sST2 combined measurements on the diagnosis and prognosis of sepsis. In this study, we collected baseline SOFA scores and basic physicochemical data of 91 hospitalized patients with sepsis. Subsequently, sTREM-1 and sST2 tests were performed to analyze the correlation between markers and SOFA scores. Next a prospective cohort study of sTREM-1 combined with sST2 was conducted to predict the prognosis of patients with sepsis. sTREM-1 and sST2 tests were performed 1, 3, 5, and 7 days after admission to analyze the prognostic value of SOFA baseline score and post-admission indicators for sepsis.

## 2 Methods

### 2.1 Patients

First, patients who meet the requirements are screened according to the following criteria.

Inclusion criteria: age ≥18 years and <75 years; suspected infection and qSOFA score ≥2 [20]; and the subject or agent understands and signs the informed consent.

Exclusion criteria: history of human immunodeficiency virus infection; history of autoimmune disease; history of malignancy; serious cardiovascular and cerebrovascular diseases (such as stroke and myocardial infarction); use of glucocorticoids and other immune-affecting drugs within 3 months; has participated in or is participating in other clinical studies within 3 months; and pregnant or lactating woman.

According to the above criteria, 91 patients with sepsis admitted to intensive care units of our hospital from May 2021 to October 2022 were selected. sST2 and sTREM-1 data were collected on the first, third, fifth, and seventh day of admission. The patients’ survival was followed up for 60 days. The groups of death and survival were distinguished according to the patients’ prognostic outcomes.


**Informed consent:** Informed consent has been obtained from all individuals included in this study.
**Ethical approval:** The research related to human use has been complied with all the relevant national regulations, institutional policies, and in accordance with the tenets of the Helsinki Declaration, and has been approved by Experimental Ethics Committee of the Second Hospital of Tianjin First Central Hospital.

### 2.2 Sample testing

On the first, third, fifth, and seventh day of admission, 2 ml of peripheral venous blood was collected on an empty stomach, and the upper serum was taken by centrifugation at 3,000 rpm for 10 min. The upper serum was collected within 4 h for detection. If the blood sample was not detected in time, the upper serum could be taken after centrifugation and stored in the refrigerator at −80℃, which could only be used once after freezing. sTREM-1 was detected using an enzyme-linked immunosorbent assay (ELISA) kit (Abcam commercial test, Cambridge, MA, USA). sST2 was detected by Beijing Keyingmei Human Stromelysin-2 ELISA kit. The operation process is carried out by professional inspectors in strict accordance with the test kit instructions and instrument operation requirements. All sample tests were repeated three times to eliminate errors.

### 2.3 Statistical analysis

IBM SPSS 26.0 statistical software was used for data processing and statistical analysis. Measurement data are expressed as mean value ± variance. Statistical data were represented by the number of cases (%), and Student’s *t*-test was used for comparison between groups [21]. Spearman rank correlation was used for correlation analysis [22]. The receiver operating characteristic (ROC) curve was used to calculate the area under curve (AUC) and Youden index (the concentration of sST2 and sTREM-1 is the cut-off value when Youden index reaches its maximum value), and the predictive value of sST2 and sTREM-1 for the severity and prognosis of sepsis patients, as well as the predictive value of sST2 combined with sTREM-1 for sepsis were analyzed [18]. *p* value < 0.05 was considered statistically significant. Without special emphasis, all samples were divided into triplicate for testing.

## 3 Result

### 3.1 A comparison between the death and survival groups

**Figure 1:**
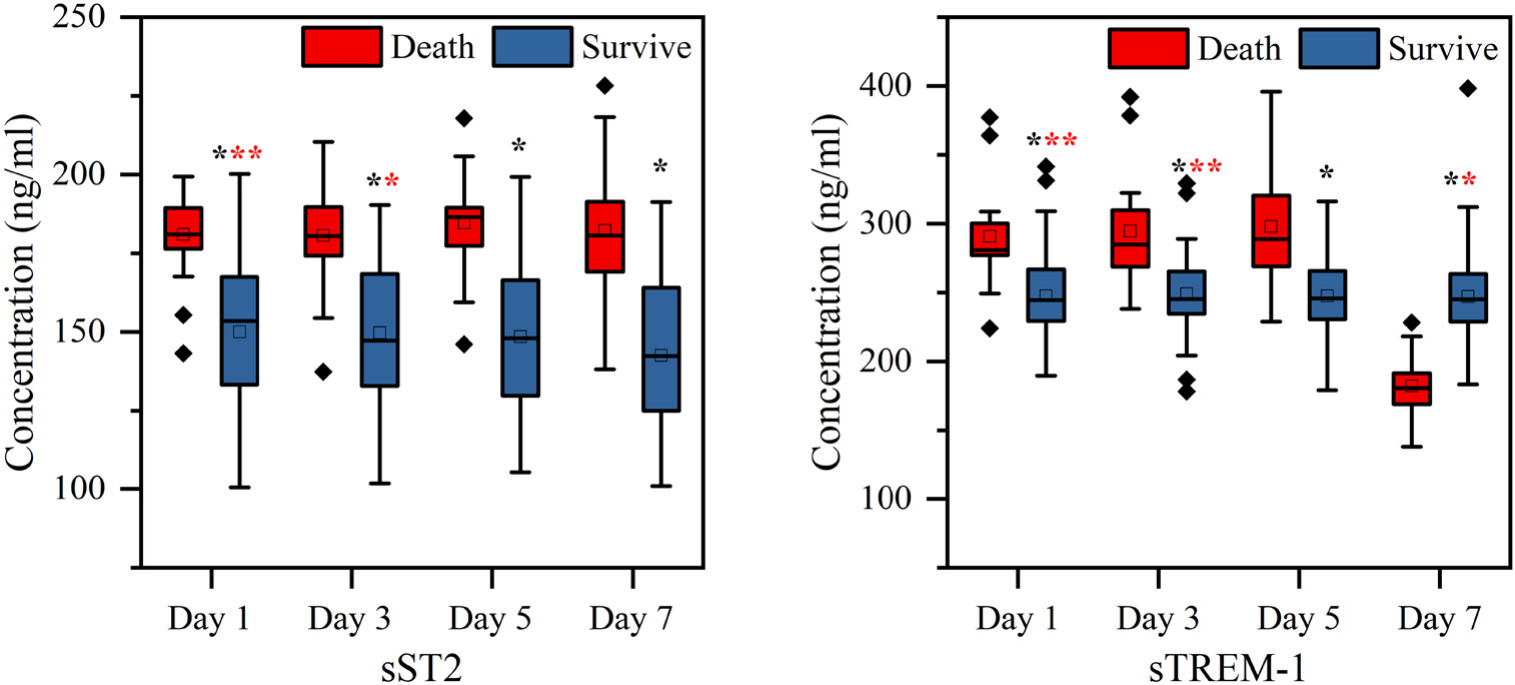
Comparison of sST2 and sTREM-1 contents between death group (*n* = 22) and survival group (*n* = 69). *p* values less than 0.05, 0.01, and 0.001 are indicated by *, **, and ***, respectively.

Herein we collected a total of 91 patients with sepsis. A total of 22 patients died (death group) and 69 patients survived (survival group, control group). Detailed information of sample characteristics is given in Table S1. sST2 and sTREM-1 contents were dynamically detected in the two groups. As shown in Figure 1, compared with the survival group, sST2, and sTREM-1 contents were significantly higher in the death group at admission to EICU, with statistical significance (*p* < 0.001). Subsequently, on the third, fifth, and seventh day after admission to EICU, sST2 in the death group was maintained at a higher level, and the content was higher than that in the survival group (*p* < 0.05). In contrast, sTREM-1 content maintained a relatively balanced level in the survival group, while in the death group, there was a large fluctuation in the 7 consecutive days of detection. In addition, the death group also showed a higher sTREM-1 content than the survival group during the first 5 days (*p* < 0.05), but the opposite trend was observed on the seventh day (*p* < 0.01).

### 3.2 Correlation between sTREM-1, sST2, combined diagnostic factor (CDF), and score of each

Considering that sST2 and sTREM-1 are significantly associated with the death/survival grouping, both biomarkers have been reported to be predictive of sepsis [13,15]. Therefore, correlation analysis was conducted between sST2, sTREM-1, and their combined diagnosis and system score (Acute Physiology and Chronic Health Evaluation Ⅱ [APACHE Ⅱ] and SOFA). First, the CDF of sST2 and sTREM-1 was calculated by binary logistic regression analysis. The specific parameters obtained by binary logistic regression analysis are shown in Table 1. Then, combined with the above parameters, the specific value of CDF is calculated using equation (1).

(1) {\rm{CDF}}={\beta }_{{\rm{Death}}/{\rm{Survive}}}+{\beta }_{{\rm{sST}}2}\times {\rm{sST}}2+{\beta }_{{\rm{sTREM}}1}\times {\rm{sTREM}}1.]

**Table 1:** Binary logistic regression analysis of sST2 and sTREM-1 (*n* = 91)

	Variable in the equation						
	Day	*β* value	S.E.	Wald value	Df	*p* value	Exp (*B*)
sST2	1	0.056	0.021	7.031	1	0.008	1.057
	3	0.053	0.02	6.616	1	0.01	1.054
	5	0.079	0.026	8.83	1	0.003	1.08
	7	0.072	0.022	10.871	1	0.001	1.075
sTREM1	1	0.021	0.011	3.344	1	0.067	1.021
	3	0.022	0.012	3.27	1	0.071	1.022
	5	0.02	0.014	2.08	1	0.15	1.02
	7	0.01	0.011	0.817	1	0.366	1.01
Death/survive	1	−16.123	3.735	18.636	1	0	−
	3	−15.924	3.638	19.16	1	0	0
	5	−19.913	4.79	17.29	1	0	0
	7	−15.669	3.681	18.123	1	0	0

CDF: combined diagnostic factor.

**Figure 2:**
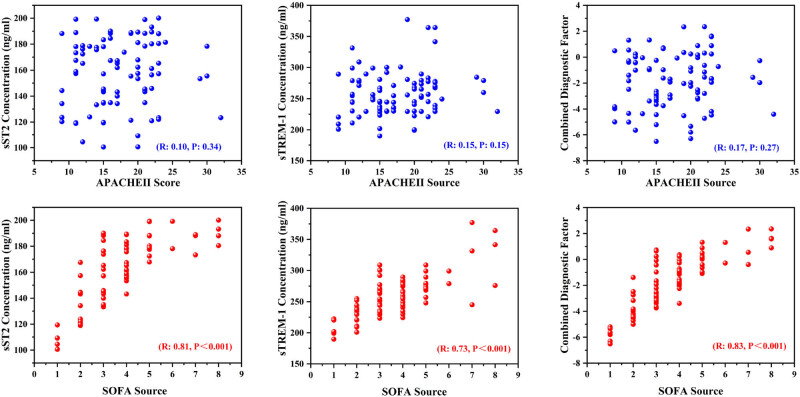
Correlation between sTREM-1, sST2, CDF, and SOFA/APACHE Ⅱ scores (*n* = 91). Using Spearman correlation test, correlation coefficient (*R*) and *p* value are given in the picture.

Then, the Spearman correlation was used to analyze the correlation between sTREM-1, sST2, CDF, and SOFA/APACHE Ⅱ scores. Correlations were analyzed for different time periods (Figure 2, Figures S3, S4, and S5) and death/survival groups (Figures S1 and S2). As shown in Figure 2, SOFA score has a higher correlation with sST2, sTREM-1, and CDF compared with APACHE Ⅱ score (*p* < 0.001). In addition, there is a higher correlation coefficient between CDF and SOFA scores under the combined diagnosis model. The results indicate that the correlation between SOFA score and joint diagnosis model may be higher, which indicates the potential advantage of combined diagnosis. To further compare the effects of death and survival grouping on the correlation between biomarkers and systematic scores, correlations between death and survival groups were analyzed (Figures S1 and S2). The results showed that sST2 in the death group had the strongest correlation with the APACHE Ⅱ score, but there was still no significant difference (*p* > 0.05). sST2 and CDF have a strong correlation with SOFA score, with significant differences (*p* < 0.05), and the *p* value of CDF is smaller. In comparison, the relationship between the APACHE Ⅱ score and biomarkers in the survival group was weak. There is a strong correlation between CDF and SOFA scores. According to the scatter plot, the distribution of sample points between CDF and SOFA shows an obvious regularity, indicating that the two are most correlated.

### 3.3 Prediction of sepsis by combination of sTREM-1 and sST2

To further verify the value of the combined diagnostic model in the diagnosis and prognosis prediction of sepsis, dynamic change data of sST2 and sTREM-1 within 7 days were obtained, and correlation analysis and ROC analysis were performed.

To evaluate the prognostic efficacy of combined diagnosis in sepsis, correlation analysis was performed between biomarker levels at days 1, 3, 5, and 7 and systematic scores (Figure 2, Figures S3, S4, and S5). The corresponding correlation coefficient (*R*) and *p* value are given in Table 2. Correlation analysis results showed that sST2, sTREM-1, and joint factors showed poor correlation with APACHE Ⅱ scores, and the *p* values of all groups were greater than 0.05. There was a weak correlation between APACHE Ⅱ scoring criteria and selected biomarkers. Meanwhile, sST2, sTREM-1, and joint factors showed a strong correlation with SOFA score (*p* < 0.001). Furthermore, the CDF group showed a higher correlation coefficient (*R*) than that of the single biomarker, and the correlation coefficient of the CDF group showed a tendency to decrease with the increase in the time of admission to the EICU.

**Table 2:** Correlation analysis of sST2, sTREM-1, and CDF with SOFA/APACHE Ⅱ score

		APACHE Ⅱ score		SOFA score	
Biomarkers	Day	*R*	*p* value	*R*	*p* value
sST2	1	0.101	0.341	0.808	<0.001
	3	0.095	0.369	0.825	<0.001
	5	0.048	0.655	0.765	<0.001
	7	0.125	0.236	0.795	<0.001
sTREM-1	1	0.154	0.145	0.725	<0.001
	3	0.140	0.186	0.775	<0.001
	5	0.094	0.377	0.757	<0.001
	7	0.118	0.267	0.712	<0.001
CDF	1	0.116	0.274	0.832	<0.001
	3	0.108	0.309	0.834	<0.001
	5	0.060	0.572	0.803	<0.001
	7	0.131	0.216	0.804	<0.001

Note: Spearman correlation analysis was used, and the sample size was 91.

**Figure 3:**
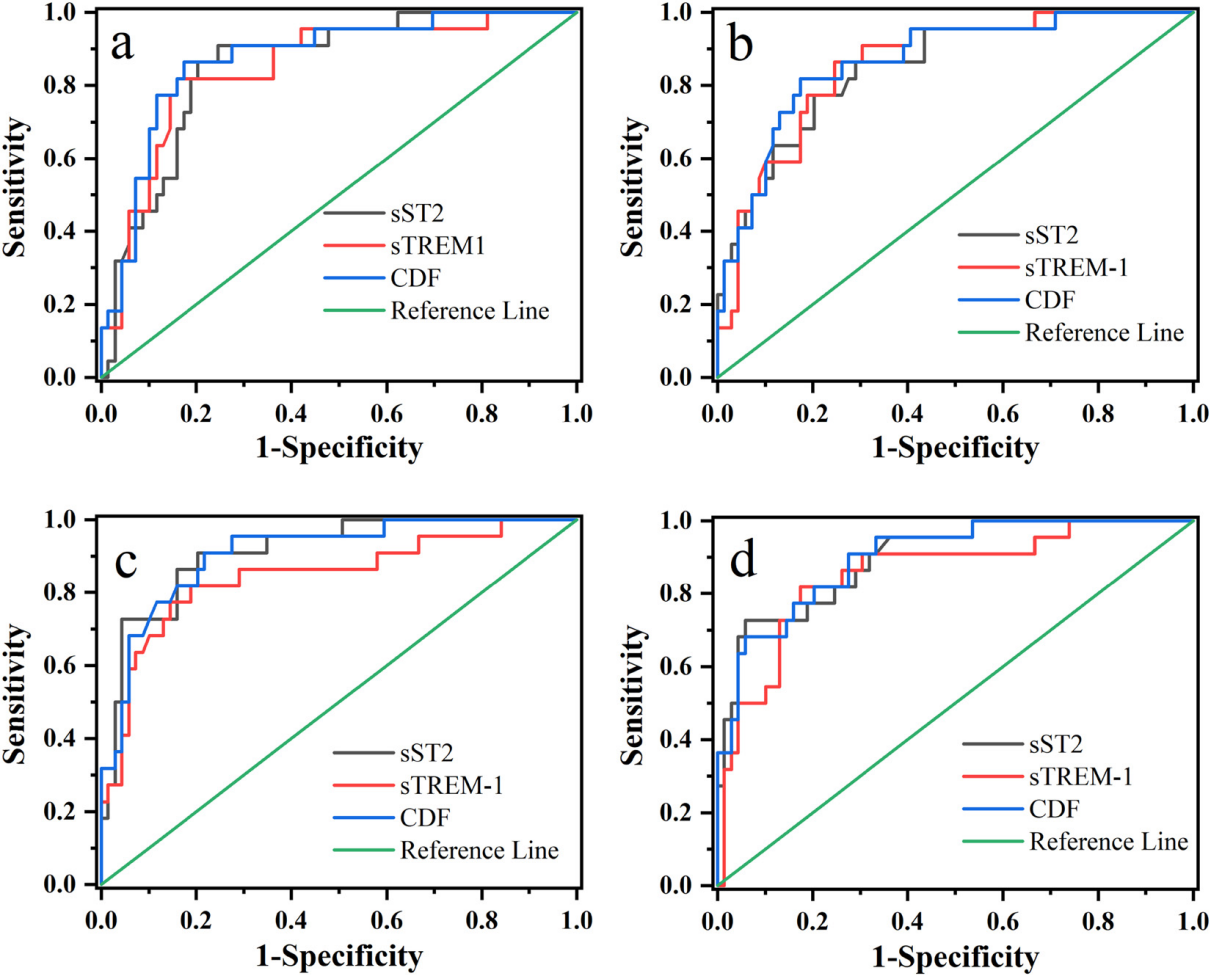
Prognostic prediction of sST2, sTREM-1, and CDFs in sepsis (*n* = 91). ROC analysis curves (a) day 1, (b) day 3, (c) day 5, and (d) day 7.

The samples were divided by death (1) and survival (0), and then ROC analysis was performed. As shown in Figure S6, the CDF group showed the largest AUC value. The results indicated that the combined diagnosis model was superior in predicting the prognosis of sepsis. To further distinguish the prognostic effect of the combined diagnostic model on sepsis under the influence of time, sST2, sTREM-1, and combined factors within a 7-day period were selected for ROC analysis. The analysis results are shown in Figure 3. The parameters obtained from the analysis are given in Table 3 and Table S1. ROC analysis results showed that CDF curves were above sST2 and sTREM-1 curves (Figure 3).

**Table 3:** AUC of ROC curve

	Day	Youden index	Cut-off concentrations (ng/ml)	AUC	Standard error	95% Confidence interval
sST2	1	0.66	167.51	0.85	0.043	0.77–0.94
	3	0.71	171.81	0.85	0.046	0.76–0.94
	5	0.57	166.28	0.91	0.033	0.85–0.98
	7	0.67	176.90	0.90	0.037	0.83–0.97
sTREM-1	1	0.66	273.94	0.85	0.048	0.76–0.94
	3	0.63	275.14	0.86	0.043	0.77–0.94
	5	0.62	265.52	0.85	0.054	0.74–0.95
	7	0.64	267.23	0.86	0.048	0.76–0.95
CDF	1	0.69	−	0.87	0.042	0.79–0.96
	3	0.69	−	0.87	0.043	0.78–0.95
	5	0.62	−	0.91	0.035	0.84–0.98
	7	0.63	−	0.90	0.036	0.83–0.97

Statistics of AUC show that, except for the fifth day, the AUC of CDF is smaller than sST2 and sTREM-1, and the other 3 days can get a larger value of AUC. The combined diagnosis is shown to have a maximum AUC value. The difference analysis of paired sample areas under the ROC curve showed that sST2, sTREM-1, and CDF had slight differences (Table S2), showing a strong correlation.

## 4 Discussion

Sepsis, a severe and life-threatening organ dysfunction caused by the host’s dysfunctional response to infection, is associated with 18–28% of deaths [1]. Mortality from sepsis has declined over time but remains stubbornly high. The best treatment options include early antibiotic treatment, resuscitation, and control of the source of microbial infection [23]. However, the pathogenic organism is isolated, cultured, identified, and then targeted. During this period, there can be a long cycle of delayed medication or drug abuse. Therefore, timely diagnosis of sepsis is the priority of treatment.

This work is based on the combined detection of two biomarkers to diagnose sepsis and predict prognosis. Our study found that compared with the survival group, the level of sST2 in the death group was higher and had a significant difference (*p* < 0.05). The level of sTREM-1 in the death group was higher in the first 5 days, but lower on the seventh day (*p* < 0.05). Meanwhile, sTREM-1 maintained a relatively stable level in patients in the survival group, but showed large fluctuations in patients in the death group (Figure 1). Growth stimulating express gene 2 (ST2) is a member of the IL super receptor family, which is mainly divided into ST2L and sST2 subtypes [24,25]. The level of sST2 was highly expressed in the death group. Compared with the traditional cardiac function index NT-proBNP, the concentration of sST2 was not only affected by myocardial problems caused by renal function and other factors, but also correlated with cardiac function grading and prognosis [26]. sST2 has been included in the 2013 ACCF/AHA Guidelines and the 2014 Chinese Guidelines for the Diagnosis and Treatment of Heart Failure [27,28,29]. As a marker related to cardiovascular diseases, a large number of studies have confirmed the correlation between sST2 and acute and chronic heart failure, myocardial infarction, myocardial fibrosis, and other diseases [30,31,32,33], but sST2 as a single marker for the diagnosis of sepsis still has deficiencies. In addition, a meta-analysis conducted by Wu et al. showed that plasma sTREM-1 had moderate diagnostic performance in differentiating sepsis from SIRS, but plasma sTREM-1 as a single marker was not sufficient for the diagnosis of sepsis in patients with systemic inflammation [34].

Clearly, the prediction of a single biomarker is inadequate, considering the differential expression of sST2 and sTREM-1 found in the death and survival groups. We hypothesize that the combined diagnosis of the two may be more effective in the diagnosis and prognosis of sepsis. Therefore, we conducted a follow-up correlation analysis and ROC analysis to evaluate the possibility of sST2 combined with sTREM-1 in the diagnosis and prognosis of sepsis. Studies have shown that combined diagnosis has advantages over single marker diagnosis in sepsis detection [35,36]. Gibot et al. found high performance in diagnosing sepsis in critically ill patients with a score that combines PMN, CD64 index, PCT, and sTREM-1 serum levels [37]. Correlation analysis showed that different from the independent sST2 and sTREM-1 single diagnosis, the combined diagnosis model had a higher correlation with SOFA score (*p* < 0.001), and the 7-day continuous detection results had a better correlation with SOFA score. ROC curve analysis results showed that compared with single biomarker diagnosis, combined diagnosis was more accurate in predicting the prognosis of sepsis (Figure 3). Statistics of ROC results showed that compared with the single biomarker diagnosis, the combined diagnosis obtained the largest AUC, indicating that combined diagnosis had relatively high accuracy. In addition, the AUC values of the combined diagnostic model increased over time from days 1 to 7, suggesting that combined diagnosis can be used to predict the prognosis of sepsis.

It is speculated that sST2 combined with sTREM-1 can be used for a more accurate diagnosis of sepsis for the following reasons: sST2 and sTREM-1 are highly expressed in the circulating blood of patients with sepsis [38,39]. When cells are damaged or necrotic, the serum expression of IL-33 increases, binds to the ST2 receptor complex, mediates the transcription of Th2 cytokines and induces an immune response [40]. sST2, as a negative regulatory factor, binds to IL-33 and participates in the immunosuppression process. At the same time, when cells are infected, the expression of TREM-1 is increased, which synergies with toll-like receptors to stimulate the production of inflammatory factors, and the expression of sTREM-1 is also increased [41]. Therefore, based on the positive response mechanism of sST2 and sTREM-1 in the immune response of sepsis, the combined diagnosis of SST2 and STREM-1 has relatively high accuracy for the detection and prognosis prediction of sepsis.

As a potential solution, the combined diagnostic model is of great significance in the diagnosis and prognosis prediction of sepsis. Our work has created a new idea for follow-up research and provided valuable research data for the further development of the gold standard of clinical diagnosis of sepsis.

## 5 Shortcomings of the research

The sample size used in this study is still insufficient, and there is a significant difference in the number of samples between the death cases of 22 and the survival group of 69. Therefore, there may be some bias in the relevant analysis. Current diagnostic strategies for clinical diagnosis of sepsis still need to be validated by sufficient study samples or machine learning. The strategy of combined diagnosis is feasible. Considering that other biomarkers may have better diagnostic effects of sepsis and prognostic value under the combined diagnosis scheme, we did not further develop these.

## 6 Conclusion

In conclusion, compared with the detection of single biomarkers, the combined detection of sTREM-1 and sST2 may be more beneficial to the diagnosis and prognosis of sepsis. Our results suggest that the combination of sTREM-1 and sST2 may be a potential choice for diagnosing sepsis and predicting its prognosis. However, it is important to note that we only examined the combined effects of sTREM-1 and sST2, and it is not excluded that other biomarkers may show the same or even superior effects in sepsis detection and prognostic prediction. Additional biomarker combinations need to be selected to evaluate the role of combined tests in sepsis diagnosis and prognosis prediction.

## Supplementary Material

Supplementary material
