# Supplementary material for: Significance of sTREM-1 and sST2 combined diagnosis for sepsis detection and prognosis prediction
Source: Open Life Sci. 2023 Aug 17;18(1):20220639. doi: 10.1515/biol-2022-0639 (PMC10436778; doi:10.1515/biol-2022-0639)
Supplement: Supplementary material [file biol-2022-0639-sm.pdf]

Supplementary material

Table S1: Basic characteristics of samples

ID	Survival/death	Gender	Age	APACHEII	SOFA	sST2 (ng/ml)				sTREM-1 (ng/ml)			
						Day 1	Day 3	Day 5	Day 7	Day 1	Day 3	Day 5	Day 7
1	Survival	Female	69	9	2	144.5	147.5	120.2	121.1	252.12	259.14	224.52	239.3
2	Survival	Female	39	8	2	143.21	140.19	133.87	130.28	234.78	245.89	244.87	245.19
3	Survival	Female	19	27	3	133.27	132.89	129.73	147.33	251.23	247.47	241.03	231.91
4	Survival	Female	64	28	2	123.34	137.23	127.33	111.33	239.11	239.42	254.29	255.54
5	Survival	Female	52	21	1	109.17	120.12	111.29	100.95	200.22	209.86	205.63	208.33
6	Survival	Female	39	6	1	119.34	118.84	109.34	101.29	201.91	204.45	208.94	199.34
7	Survival	Female	56	2	3	157.12	142.23	152.91	146.32	223.34	229.45	251.23	225.34
8	Survival	Female	72	17	3	165.34	145.82	166.43	155.74	235.66	239.06	248.04	248.93
9	Survival	Female	70	11	2	134.23	129.03	133.29	121.39	220.34	231.09	233.81	219.05
10	Survival	Female	69	7	2	144.23	119.34	128.34	122.32	200.91	231.08	229.01	261.03
11	Survival	Female	93	12	2	157.34	142.33	121.22	122.07	230.23	251.06	229.34	219.83
12	Survival	Female	87	14	4	183.44	167.33	169.83	172.34	266.89	257.34	275.96	287.36
13	Survival	Female	82	16	4	167.45	176.34	134.55	139.83	244.56	235.34	254.22	220.34
14	Survival	Female	87	16	3	184.54	163.22	132.33	122.87	229.34	210.34	209.34	200.09
15	Survival	Female	69	9	4	165.34	155.97	162.34	164	234.49	255.07	265.82	266.72
16	Survival	Female	62	14	3	143.28	165.22	155.27	152.34	265.34	242.31	247.97	251.09
17	Survival	Female	83	20	3	134.23	144.23	145.28	144.23	271.09	256.43	244.32	239.91
18	Survival	Female	28	8	2	118.86	128.07	129.76	122.07	243.97	241.74	252.83	229.54
19	Survival	Female	75	9	1	104.52	110.64	121.04	117.64	220.53	216.94	211.02	209.15
20	Survival	Female	80	12	4	175.78	165.83	166.43	167.92	256.45	256.78	266.62	257.33
21	Survival	Female	45	7	2	121.03	118.35	118.34	109.46	220.93	221.83	228.83	219.42
22	Survival	Female	64	9	6	199.23	178.33	189.36	191.25	299.01	289.03	278.91	279.04
23	Survival	Female	85	12	2	122.1	121.92	123.93	131.3	229.34	228.34	239.98	241.27
24	Survival	Female	69	17	3	139.93	142.38	144.28	142.81	244.01	241.09	248.95	255.01
25	Survival	Female	81	2	3	134.98	142.3	149.36	132.01	233.87	243.9	239.71	228.91
26	Survival	Female	69	9	5	178.92	182.32	188.09	145.08	271.04	251.03	255.91	270.91
27	Survival	Male	69	11	7	173.41	173.1	181.55	169.32	331.44	329.28	316.22	305.21
28	Survival	Male	54	23	8	188.15	187.5	179.2	172.1	341.49	329.22	307.23	398.24
29	Survival	Male	52	20	1	100.7	101.8	105.49	103.31	199.23	186.88	179.23	183.44
30	Survival	Male	78	9	4	161.23	158.33	145.34	123.34	283.22	274.88	246.78	259.92
31	Survival	Male	68	6	3	157.24	155.22	157.34	131.22	267.34	266.29	255.37	247.63
32	Survival	Male	59	22	3	145.89	147.23	156.24	144.01	245.38	248.33	228.91	231.29
33	Survival	Male	51	34	3	144.39	147.37	123.79	131.77	244.18	257.38	227.28	218.89

(Continued)

Table S1: *Continued*

ID	Survival/death	Gender	Age	APACHEII	SOFA	sST2 (ng/ml)				sTREM-1 (ng/ml)			
						Day 1	Day 3	Day 5	Day 7	Day 1	Day 3	Day 5	Day 7
34	Survival	Male	24	20	4	165.23	177.73	167.34	157.33	279.39	278.93	291.23	268.17
35	Survival	Male	56	8	3	157.23	156.37	137.33	139.72	269.86	271.27	274.36	266.18
36	Survival	Male	30	38	5	172.47	177.23	166.32	161.93	308.91	322.09	300.91	312.09
37	Survival	Male	52	85	1	100.53	109.22	105.28	107.02	189.72	178.34	188.92	200.91
38	Survival	Male	72	20	4	181.23	177.34	183.22	167.23	245.23	244.51	231.49	233.45
39	Survival	Male	67	11	3	162.23	147.39	133.87	127.34	229.83	234.59	219.83	222.34
40	Survival	Male	65	15	2	123.56	134.34	139.91	121.84	200.81	223.94	233.91	234.87
41	Survival	Male	70	11	2	119.45	132.45	133.27	129.67	210.93	230.84	227.91	245.01
42	Survival	Male	53	17	4	167.35	147.55	155.86	152.94	229.93	246.96	249.65	252.07
43	Survival	Male	64	10	4	157.45	173.45	155.34	149.89	240.66	261.45	271.45	288.74
44	Survival	Male	77	20	5	167.84	177.32	171.32	176.45	268.45	278.45	288.34	266.45
45	Survival	Male	58	10	4	153.45	168.46	166.34	143.23	265.34	255.31	247.29	238.45
46	Survival	Male	86	5	4	153.45	173.45	163.44	172.44	284.55	265.44	254.38	248.39
47	Survival	Male	82	17	3	134.58	139.03	148.03	127.92	263.03	229.49	238.09	231.44
48	Survival	Male	73	15	4	155.49	157.34	154.23	173.44	260.01	244.97	261.03	284.55
49	Survival	Male	74	64	5	188.34	167.34	166.74	169.32	273.43	266.41	256.73	247.83
50	Survival	Male	63	32	2	123.34	118.99	123.95	133.22	229.45	210.44	221.09	209.94
51	Survival	Male	69	11	7	188.93	190.33	178.34	177.93	245.09	278.24	279.34	265.21
52	Survival	Male	54	23	8	200.23	180.23	178.23	167.92	276.08	289.07	267.97	277.97
53	Survival	Male	52	20	1	109.34	119.34	121.63	111.09	222.34	234.67	212.98	228.87
54	Survival	Male	78	9	5	180.34	176.09	168.82	172.83	256.88	274.32	278.93	256.43
55	Survival	Male	68	6	3	144.32	143.53	137.34	131.02	229.08	245.34	230.75	251.37
56	Survival	Male	72	16	2	120.34	112.08	123.45	110.93	209.09	213.03	234.21	221.01
57	Survival	Male	65	16	6	178.23	183.44	182.83	179.92	278.97	267.83	277.31	269.86
58	Survival	Male	88	5	4	156.34	157.38	166.34	153.97	277.65	265.32	245.75	255.45
59	Survival	Male	77	34	2	121.34	119.44	131.98	127.52	229.34	234.72	244.23	237.91
60	Survival	Male	89	7	3	134.87	141.29	165.23	142.38	254.34	243.08	238.04	239.85
61	Survival	Male	94	10	4	158.94	165.43	158.93	167.53	256.98	277.63	276.42	265.96
62	Survival	Male	56	6	5	177.65	189.04	178.42	185.49	247.99	267.52	276.53	277.85
63	Survival	Male	76	12	2	123.97	127.74	130.92	124.94	229.55	235.95	237.52	225.19
64	Survival	Male	64	46	3	145.43	144.03	132.52	143.32	245.64	242.34	237.87	263.53
65	Survival	Male	67	5	5	188.21	189.22	199.23	176.22	289.33	281.92	278.34	259.17
66	Survival	Male	89	10	3	145.44	144.26	138.92	152.34	229.34	234.89	241.2	241.38
67	Survival	Male	62	8	3	134.87	139.76	128.97	129.89	230.09	239.97	233.86	245.21
68	Survival	Male	20	17	4	161.97	145.87	155.09	132.87	254.76	255.61	260.01	238.92
69	Survival	Male	68	15	2	119.73	118.21	182.34	127.48	237.34	238.01	219.23	231.98
70	Death	Female	75	15	4	143.23	137.41	146.14	138.11	224.23	238.23	227.16	232.47
71	Death	Female	52	13	4	178.34	182.45	184.73	189.36	289.73	281.78	288.91	298.81
72	Death	Female	61	33	3	188.34	192.33	188.31	178.34	300.27	312.33	318.23	320.08

(Continued)

Table S1: *Continued*

ID	Survival/death	Gender	Age	APACHEII	SOFA	sST2 (ng/ml)				sTREM-1 (ng/ml)			
						Day 1	Day 3	Day 5	Day 7	Day 1	Day 3	Day 5	Day 7
73	Death	Female	83	4	4	178.03	189.05	188.36	193.45	280.91	268.94	275.92	277.04
74	Death	Female	82	16	3	190.09	200.07	184.23	179.85	291.95	259.07	240.34	229.04
75	Death	Female	64	19	5	199.34	210.34	200.91	189.83	299.03	278.93	300.28	310.34
76	Death	Female	77	10	4	189.44	167.34	172.46	169.08	277.34	287.98	288.91	278.69
77	Death	Female	79	3	4	155.43	154.45	159.44	166.23	255.99	265.98	276.58	277.01
78	Death	Female	69	1	5	199.02	178.39	188.27	178.39	279.29	289.97	287.92	299.01
79	Death	Female	77	9	8	193.33	187.47	199.34	218.23	364.07	378.51	377.91	376.98
80	Death	Male	67	23	3	173.84	176.61	189.43	201.63	300.76	309.87	311.98	322.93
81	Death	Male	29	58	3	176.38	189.44	190.38	191.4	308.87	322.45	333.78	321.12
82	Death	Male	85	2	4	178.49	177.97	183.45	188.32	289.65	271.91	277.98	269.09
83	Death	Male	80	35	5	188.04	194.04	188.45	178.34	277.65	271.44	258.09	263.41
84	Death	Male	67	15	4	188.94	210.23	189.34	181.49	280.84	271.34	278.93	277.63
85	Death	Male	93	20	4	189.34	174.23	172.3	153.92	274.45	289.31	299.01	300.45
86	Death	Male	77	1	4	181.44	174.94	183.49	177.34	249.44	289.54	300.45	320.45
87	Death	Male	51	2	4	178.45	178.09	184.55	189.34	279.04	289.84	288.91	267.74
88	Death	Male	75	5	3	176.34	166.73	168.74	156.37	277.23	265.59	289.34	275.34
89	Death	Male	71	10	2	167.56	154.56	177.34	152.34	254.83	254.84	234.73	257.73
90	Death	Male	71	13	8	180.57	188.14	205.79	210.77	364.07	391.87	382.44	395.94
91	Death	Male	63	10	7	188.23	189.67	217.89	228.22	377.14	392.12	389.34	387.49

Table S2: Paired sample area difference statistics under ROC curve

	Day	*Z*	Sig. (Double tail)	AUC variance	Standard error	95% CI
sST2 - sTREM1	1	0.135	0.892	0.005	0.296	−0.071 to 0.082
	3	−0.213	0.831	−0.007	0.292	−0.074 to 0.095
	5	1.454	0.146	0.066	0.292	−0.023 to 0.155
	7	0.983	0.326	0.043	0.288	−0.043 to 0.128
sST2 – CDF	1	−0.943	0.345	−0.019	0.286	−0.06 to 0.021
	3	−1.014	0.311	−0.016	0.292	−0.046 to 0.015
	5	0.272	0.786	0.005	0.257	−0.031 to 0.041
	7	−0.147	0.883	−0.001	0.264	−0.019 to 0.016
sTREM1 - CDF	1	−1.077	0.282	−0.025	0.294	−0.07 to 0.02
	3	−0.376	0.707	−0.009	0.288	−0.053 to 0.063
	5	−2.039	0.041	−0.061	0.292	−0.12 to −0.002
	7	−1.203	0.229	−0.044	0.286	−0.116 to 0.028

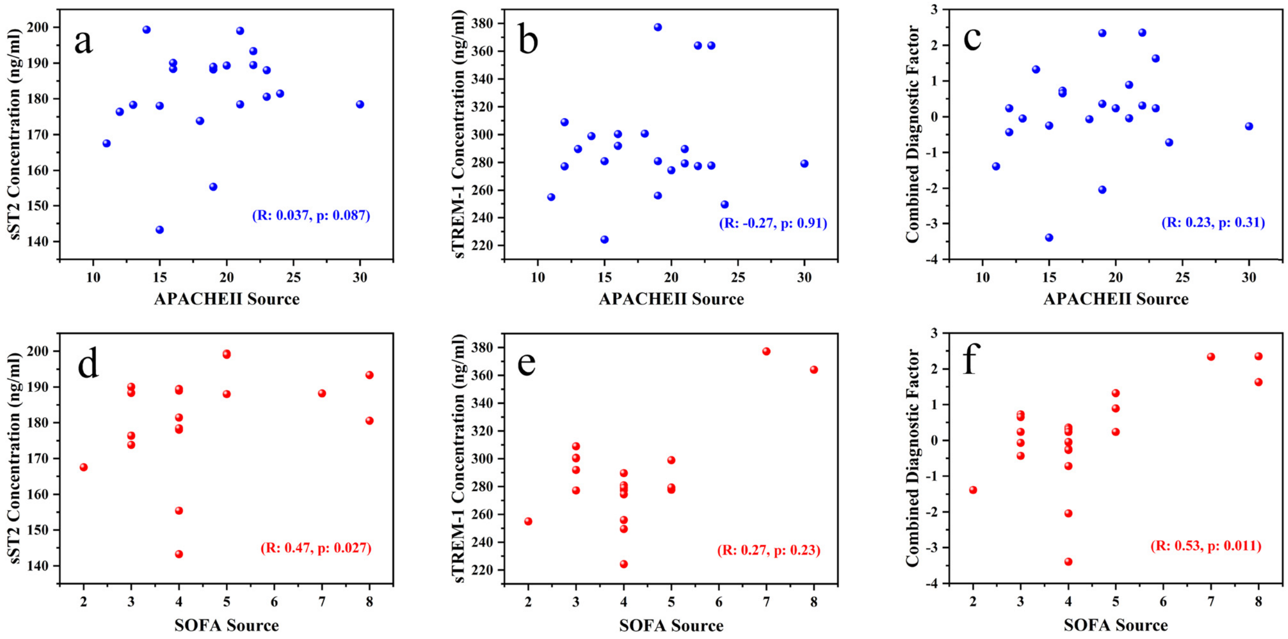

Figure S1: In death group: Correlation between sTREM-1, sST2, combined diagnostic protocol, and SOFA/APACHE II score (n = 22). Using Spearman correlation test, correlation coefficient and p value are given in the picture.

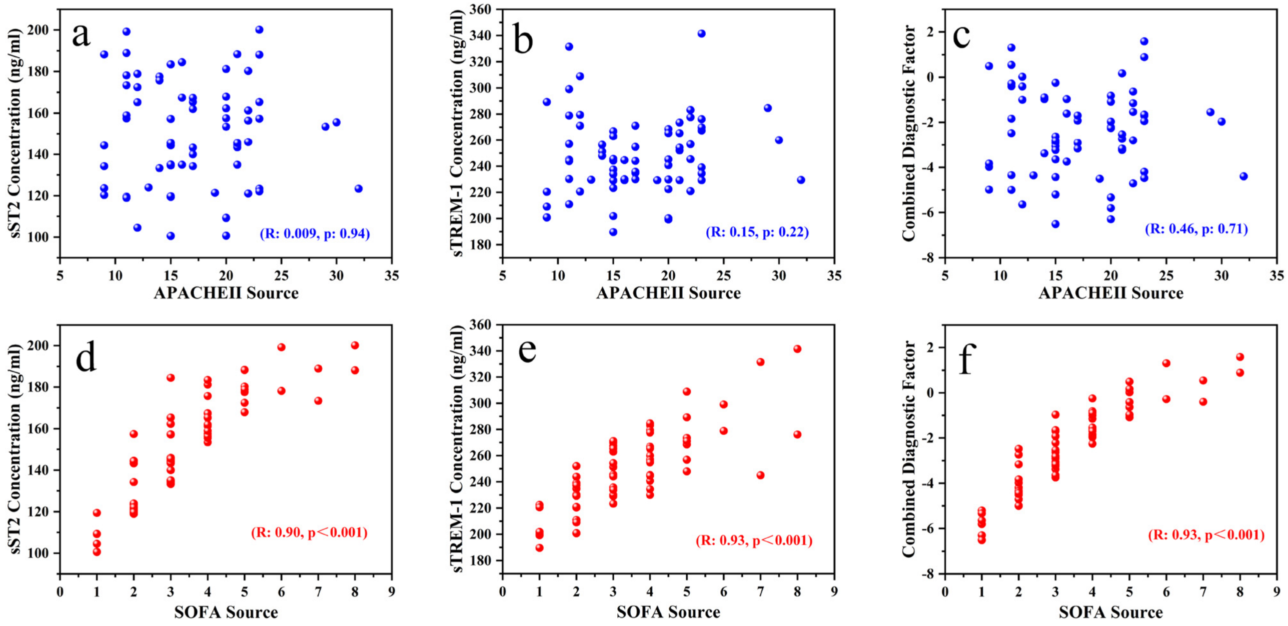

Figure S2: Survival group: Correlation between sTREM-1, sST2, combined diagnostic protocol, and SOFA/APACHE II score (n = 69). Using Spearman correlation test, correlation coefficient and p value are given in the picture.

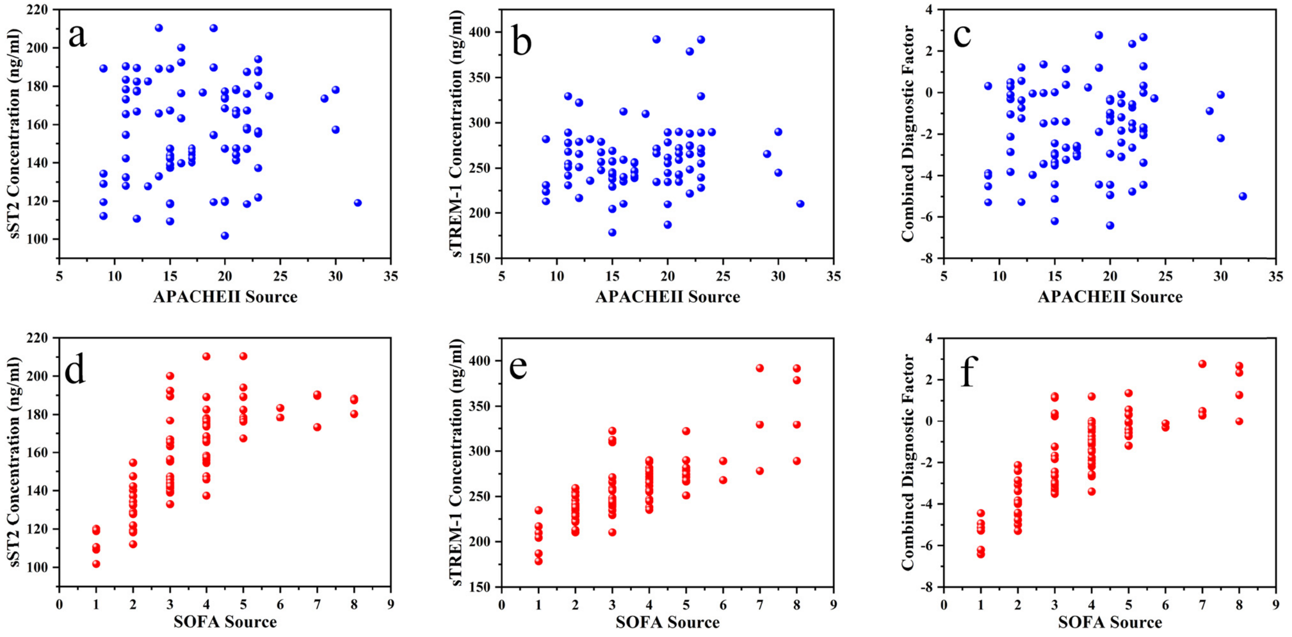

Figure S3: Correlation analysis of SOFA/APACHE II scores by sST2, sTREM-1, and combined diagnostic protocol on the third day. Spearman correlation analysis was used, and the sample size was 91.

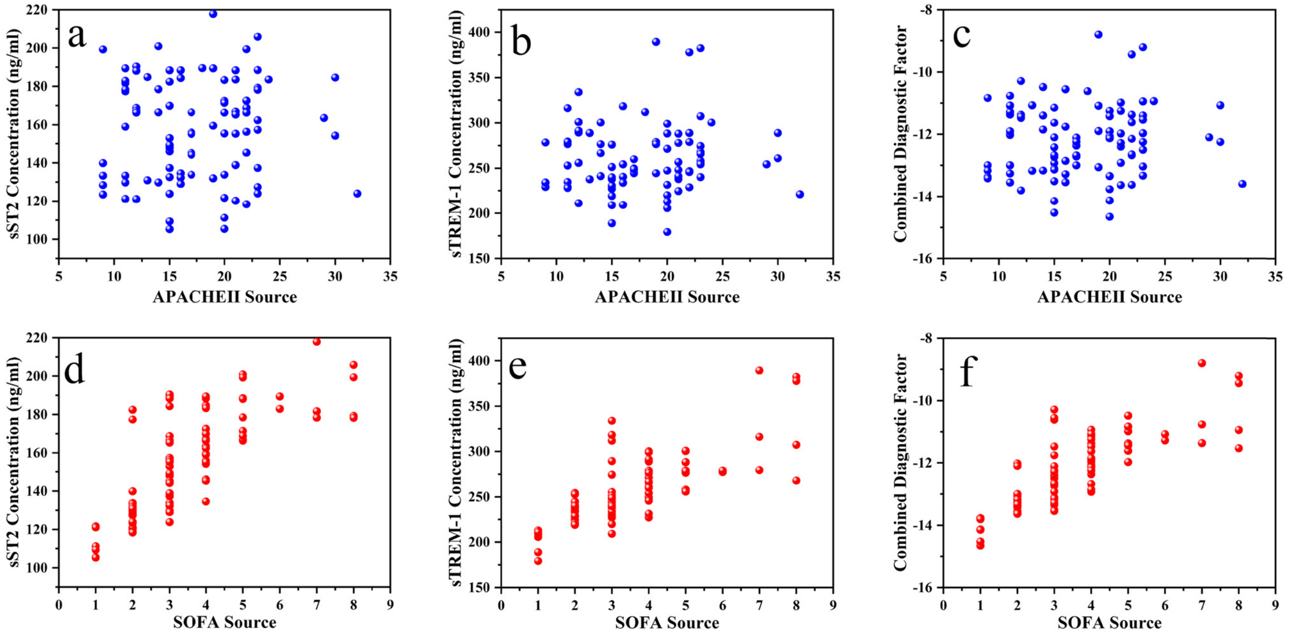

Figure S4: Correlation analysis of SOFA/APACHE II scores by sST2, sTREM-1, and combined diagnostic protocol on the fifth day. Spearman correlation analysis was used, and the sample size was 91.

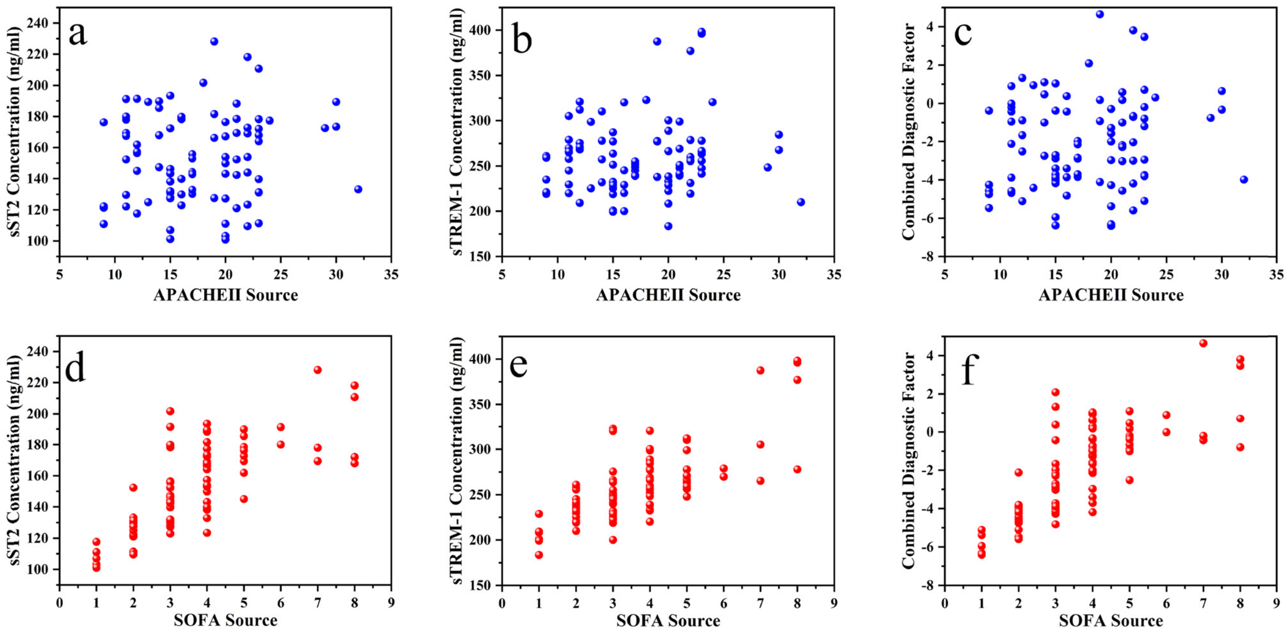

Figure S5: Correlation analysis of SOFA/APACHE II scores by sST2, sTREM-1, and combined diagnostic protocol on the seventh day. Spearman correlation analysis was used, and the sample size was 91.

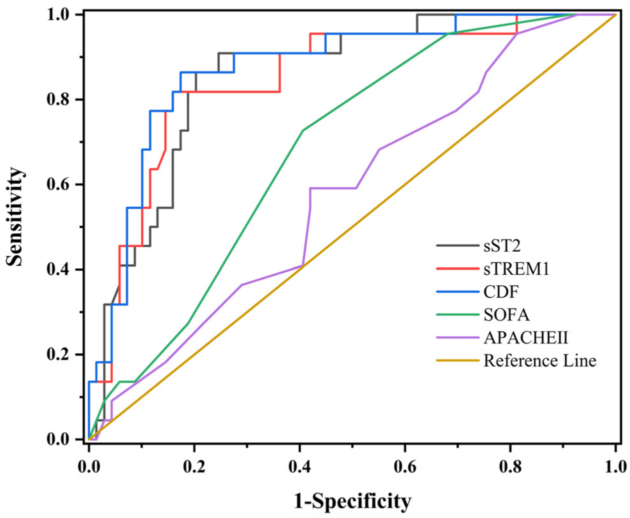

Figure S6: ROC curve of sST2, sTREM-1, and combined diagnostic factors for prognostic effects of sepsis (n = 91).
